# Mr. Ward, on Alcohol, &c.

**Published:** 1803-01-01

**Authors:** M. Ward

**Affiliations:** Manchester


### *To the Editors of the Medical and Physical Journal.*

GENTLEMEN,

I AM happy to find your anonymous Correspondent, whose letter appears in your last Number, Vol. VIII. p. 505, is inclined to adopt my opinion of the sedative properties of opium, for if our ideas coincide on this great and leading question, other differences will be easily adjusted, or will be of no real importance.

"I am inclined," (says he) "too, to adopt his opinion of the sedative properties of opium; but cannot help thinking that, in his investigation of its modus operandi, (a question in the highest degree recondite, and perhaps of very little practical utility) he has been led into many hasty assertions, and has drawn some unfounded conclusions. His censures of Dr. CRUMPE's Treatise appear unwarrantably severe, and needed needed not the additional aid of *italics* and *capitals* to render them more pointed."

The first charge of having been led into some hasty assertions, may possibly be well founded, because the plan I have adopted of publishing a series of connected observations in detached parts, is attended with some disadvantages; and I have not yet examined the different papers with that attention it would require to detect them, if there be any: but I am not aware of that being the case, and am still less conscious of having drawn any unfounded conclusions, or of having been unwarrantably severe in my censures of Dr. CRUMPE's Treatise. I shall therefore avail myself of the privilege allowed in all courts, of pleading not guilty, till some proofs are brought in aid of the accusations. How far your correspondent has avoided those errors, which, without adducing one single proof, he has attributed to me, remains to be seen.

With regard to the modus operandi of opium being a question in the highest degree recondite; the terms in which I have expressed myself in the different pages of your Journal, (see Vol. VII. p. 355—358, 497—506; Vol. VIII. p. 330) render it both improper and uneccessary for me to enter into it as an abstract question.

So far am I from agreeing with him in thinking it a question of very little practical utility, I am of opinion, that if there be a physiological question which involves in it more momentous consequences than any other, it is that which relates to the modus operandi of opium.

Can it be supposed, that a question which has engaged the attention of philosophers and physicians, in almost every age and country, since opium became known as a medicine, is of very little practical utility? A question which has exercised the skill and ingenuity of men so celebrated as Drs. Mead, Alston, Whytt, Monro, Langrish, Cullen, Johnstone, Young, Darwin, Brown, and Heberden, in our own country; to which a long list of men, equally famous for knowledge and skill, in other countries, may be added, such as Tralles, Fontana, &c. &c. Nor would it be just, on the present occasion, to omit the names of Drs. Currie, Alexander, and Wilson, (the two latter of whom, in particular, have added greatly to our stock of knowledge, by the publication of their ingenious and accurate experiments on opium). Can it be supposed, I say, that these men, and a great number besides that I am unacquainted with, have been wasting their time and talents upon a question of very little practical utility? Such an idea would, in my opinion, be absurd. Indeed, it is only necessary to consult the works of the different writers who have favoured the public with their sentiments on the subject, and especially those of a recent date, to be convinced of the urgent necessity of its being ascertained by the fullest and fairest investigation, whether opium operates as a stimulant, or directly as a sedative.

One consequence of its being considered as a stimulant, is, that all those substances which, like opium, immediately diminish and ultimately destroy the sensibility, irritability, and mobility of the body, such as cicuta, belladona, hyoscyamus, nicotiana, lauro cerasus, camphora, æther, volatile alkali, musk, thea, alcohol, &c. &c. are considered by many writers as stimulants also;* a fact which, I think, fully demonstrates the extreme importance of the question.

There are other points to which I must now beg leave to call your attention. C. C. W. proceeds as follows:

"In his last letter, Mr. Ward has himself advanced an assertion, which appears, *prima facie*, most glaringly false and untenable: namely, that alcohol is a sedative. Now, the purport of this letter is, to request that gentleman to state the arguments which he considers as corroborative of his opinion."

Would any one who reads the above passage, suppose that I had already advanced either arguments or proofs in support of the assertion? I think not.

I find however, upon referring to vol. vii. p. 127, that I have made use of the following arguments.

"Another inaccuracy they seem to have fallen into is, their taking it for granted that wine and alcohol are stimulants, and reasoning upon that supposition, though this is also a disputed point; and Dr. Cullen, after a close investigation, places them in the class of narcotic sedatives."

The following quotations then appear in a Note:

"It is, however, as containing alcohol, that wines are to be considered as medicines; and the considering them as such, we have reserved for this place, in which I have put them as narcotic sedatives. That alcohol is such, can hardly be doubted; as, when only diluted with water so much that it can be swallowed, it shows the inebriating, intoxicating,

* Darwin's Zoonomia, vol. ii. p. 678, 692, 693. Brown's Elements of Med. vol. i. p. 103, 104; vol. ii. p. 6. Beddoes's Hygeia, No. viii. p. 26. Med. and Ph. Journal, vol. vii. p. 525, 526; vol. viii. p. 50, 53. Noble on Ophthalmia, Part ii. p. 154, 188, 217, 218.

intoxicating, and narcotic effects of other sedatives. When taken in small quantity, and much diluted, it does not indeed immediately show its sedative power; but, on the contrary, it may appear as a stimulant, cordial, and exhilirating liquor. As these operations, however, are in common to it with opium and other narcotics, they do not contradict our opinion of its proper sedative nature." Cullen's Mat. Med. vol. ii. p. 315, 16.

Alluding to wine, he says, p. 317, "It is difficult, however, to set the limits between its stimulant and sedative powers; and if the quantity of it be gradually increased, the latter gradually come on, and concurring with the former, produce at first a degree of delirium or ebriety, which is generally of the cheerful kind, and which occupying the mind, excludes all thoughts of care or anxiety; but the same sedative power, carried on still further, renders the delirium more considerable, and gives that irregularity and confusion of thought, which is the state of intoxication; and at length, the sedative power entirely prevailing, the animal functions, both of sense and motion, are gradually weakened, and the person falls asleep."

Upon referring to vol. viii. p. 339, 340, 344, 345, I find that I have related eight experiments also; three of which are taken from Dr. Crumpe's Treatise, and five from Dr. Monro's Paper on Opium, &c. in the Ess. and Obs. P. and L. vol. iii. They are as follow:

Exp. 34. "Employing rectified spirit of wine in the same manner as the other articles (namely opium and spirit of hartshorn) in the two preceding experiments, I found it succeeded,* by similar consequences, in three minutes."

Exp. 38. "Rectified spirit of wine deprived a third of all power of motion in three minutes."†

Exp. 40. "Having extended the limbs, and tied down a large frog on its back, I opened the thorax just so as to bring the heart into view; its pulsations were 62 in a minute. I then injected into the anus, by means of a small syringe, some rectified spirit of wine; but the quantity of which could not be exactly determined, as some of what had been injected was again discharged. Leaving the animal in this situation, I attended to the changes induced in the heart's motion; and reckoning its pulses at the periods mentioned

---

* In depriving the limb of all sensibility and power of motion.

† The heart of a frog separated from the body, was employed in this experiment.

in the first line of the following table, found their number to be as is set down in the second:

| | | | | | | | | | | | | | |
|---|---|---|---|---|---|---|---|---|---|---|---|---|---|
| In — | 2 | 5 | 10 | 15 | 20 | 25 | 30 | 35 | 40 | 45 | 50 | 55 | 60 |
| The P. was | 62 | 62 | 62 | 52 | 46 | 44 | 42 | 40 | 36 | 36 | 34 | 32 | 32 |

"Sixty minutes elapsed, the animal was in external appearance dead, although the heart still continued to beat, and was thrown out."

The above experiments are taken from Dr. C's Inquiry. The five which follow, from Dr. Monro's Essay.

( Four Trials. )

"About seventy drops of a mixture of equal parts of white wine and French brandy were applied on lint to the hind extremities of a frog. In seven minutes the animal was observably affected, and its heart beat only fifty times in a minute. In a quarter of an hour, the heart beat only forty-five times in a minute; yet the rapidity of the blood in the hind feet was not very observably lessened. After twenty minutes, the heart beat only forty times in a minute, and the creature was much stupefied. After half an hour, the heart beat only thirty-five times in a minute, and evidently with less force than natural; now the circulation was considerably less rapid in the vessels of the hind feet, and the creature was scarcely able to withdraw its legs when the toes were pinched. After three quarters of an hour, the heart contracted but thirty times in a minute, and the motion of the blood in the vessels of the hind feet was extremely slow, and ceased altogether after fifty-five minutes, at which time the creature was unable to withdraw its legs from the most severe injury; or when the eye ball was fretted with a probe, to shut its eyes, which were wide open; and its respiration had entirely ceased. I now removed the mixture and washed the legs in water; notwithstanding which, it remained insensible for about an hour and a half longer, although the heart continued the whole time to vibrate, feebly indeed, about thirty times in a minute. But after this it began to bend the joints of the hind legs spontaneously; and in forty minutes more was able to scramble over the edge of the plate on which it was laid; yet, till half an hour after, when the heart contracted forty times in a minute, the progressive motion of the blood in the feet was not renewed. Thus, the animal gradually recovered its sensibility and motion, and the blood its free circulation; after three hours more, or about the end end of the seventh hour from the beginning of the experiment, it could jump with almost its wonted vigour, and the motion of the blood in the feet appeared free and rapid.

When the mixture was applied to one leg only, that leg seemed to be somewhat, but not very considerably, more benumbed than the other; yet the circulation did not cease sooner in the one than in the other leg." Ess. p. 340, 342. " Compare Dr. Alexander's 20th, 21st, 22d, and 23d Experiments.

As C. C. W. asks for farther proofs, I will introduce the Experiments to which I have referred.

EXPERIMENT 20.

H. M.
5 0 Sterno ranæ adultæ sedulo ablato, corde exscisso, quinquaginta guttæ solutionis potentis (opii) in ventriculum, vitreo syringe, injiciebantur; complures evasere.
0 15 Animal haud parum stupefactum; extrema extensa et fere paralytica videbantur.
0 20 Animal convellebatur. Cultri acie extremis punctis, sensatio excitatur.
0 40 Facultas voluntarii motus penitus extincta; at, ubi contactum est, convellebatur.
6 10 Animal mortuum. Nihil motus aut sensationis excitari potest.

Musculis denudatis, stimulus frustra adhibebatur; nervo crurali diviso, et deinde compresso, nihil contractionis sequebatur.

EXPERIMENT 21.

Triginta ejusdem solutionis guttæ, in ventriculum alius ranæ injiciebantur, corde antea remoto, pariter ut proxima convellebatur, et paulo post horam et quindecim minuta, e vita excessit. Post mortem nihil vis contractilis ulli musculorum omnino superfuit.

EXPERIMENT 22.

Viginti guttæ in ventriculum alius ranæ injiciebantur. Hora duodecimque minutis elapsis, animal mortuum est. Post mortem nulla vis contractilis signa musculi exhibuere.

EXPERIMENT 23.

H. M.

6 45 Quinquaginta guttis *spt. vin. rect.* in ranæ ventriculum injectis, corde ut supra remoto.

Animal

H. M.
7 0 Animal stupefactum, et lethargicum; at stimulatum, sensationem adhuc retinet.
0 50 Animal moritur. Musculis nudatis, et irritatis, et crurales nervos premendo, et stimulum musculis adhibendo, nihil contractionis excitabatur.

Hinc deducendum,

1mo. Spiritus vini rect. et opii in ventriculum injectionem, corpora ranarum, quarum corda remota fuerant, pariter afficere.

2do. Partes remotas æque ac partem cui injectio adhibeatur, affectas esse.

3tio. Hac operatione, vim contractilem fibræ mnscularis deleri.

4to. Nec ad hoc efficiendum, circulationis auxilio esse opus. Dissert. Inaug. de Opio. p. 28—31.

The experiments (twelve in number) and arguments above transcribed, prove satisfactorily to my mind, that alcohol acts directly as a sedative: but, at any rate, they prove, (which is sufficient for the present purpose) when compared with the preceding and subsequent experiments, and taken in conjunction with a great number of remarks, quotations, and references, which occur in the different papers on this subject inserted in your Journal, that it acts in the same manner as opium, (indeed this is a point which has long been settled) and C. C. W. expressly says in his letter, "I am inclined to adopt his (Mr. Ward's) opinion of the sedative properties of opium:" he must, therefore, either admit that alcohol is a sedative, or it will be incumbent npon him to shew, that its modus operandi is different to that of opium.

Now, I contend that the arguments and experiments related above, and which had been given before, or referred to, are so numerous and demonstrative, that it is impossible to consider them in the light of *prima facie* evidence; we must therefore suppose they had escaped the notice of your Correspondent.

The following passage in my last paper (vol. viii. p. 392) will clearly prove that I did not by any means consider them as deciding the question.

"It is allowed on all hands, that alcohol operates in the same manner as opium; and I shall now consider it as an established fact, that the mod. op. of the latter, and of course of the former, is directly sedative." And, in a note, I have said (and to this I wish particularly to call your attention)

tention) "Farther proofs may be thought necessary, and these I shall endeavour to supply in a succeeding paper."

This promise I always intended, and still intend, to perform. But I must now go on to reply to the concluding paragraph in C. C. W's letter. It is as follows:

"But that we may not dispute about terms, I would likewise beg the favour of him (Mr. Ward) to give his definition of the terms stimulant and sedative: for if the former term be applied, as it most commonly is, to certain substances which excite, for a time, the action of the heart and arteries, increase muscular power, and the sensorial functions, I do not perceive how he can possibly exclude alcohol from the list, unless, indeed, he consider the state of debility consequent on drunkenness as a proof of its possessing a direct sedative power; if so, his judgment is, I apprehend, widely different from that of other physiologists."

It plainly appears from this passage, that your Correspondent has a short memory, and so far has a claim to be considered as a great wit: (for candour forbids the idea that he would censure my remarks as hasty, unfounded, and unwarrantably severe, without having previously perused them.)

In support of the assertion, I beg leave to refer you to vol. vii. p. 127, 128, where I find I have expressed myself in the following manner:

"The subject seems to have been rendered more intricate than it really is, by the advocates for the stimulant doctrine not adhering to a precise definition of the terms, stimulants and sedatives.

"The following definitions appear to me to be unexceptionable; and I must beg to be understood to allude to them, whenever I make use of either of those terms."

"This then is the general idea of stimulants, that they are powers capable of increasing the mobility, and of exciting the motion of the nervous power." Cullen's Mat. Med. vol. ii. p. 131.

"Sedatives. These are the medicines which diminish the sensibility and irritability of the system, and thereby the motions and powers of motion in it."* Idem. p. 217.

Adhering

---

* The Editors request those Correspondents, who may be disposed to favour them with their sentiments on this difficult subject, to give their names at length, as Mr. Ward has done. They take the liberty also, of reminding their friends, that no disputed point can be adjusted while the Disputants are employed

Adhering to my original definition (or rather to Dr. Cullen's definition) of the term stimulant, which it will be easy to shew is far preferable to that your Correspondent seems inclined to adopt; I do not see that it is at all necessary, because I exclude alcohol from the list of stimulants, that I should consider the state of debility consequent on drunkenness as a proof of its possessing a directly sedative power. The idea I have always annexed to a substance acting directly as a sedative, (and I have always supposed both opium and alcohol to act in this manner) is, that it immediately diminishes the sensibility and mobility of the system, without these effects being preceded by any symptom of increased excitement, and have more than once expressed myself in words to that effect (see vol. viii. p. 336, 337); and we have only to turn our attention to the experiments above related, to be convinced that this is the mode of operation of alcohol.

A similar inference may be drawn from the following experiments of Fontana.

"Leeches immersed in spirit of wine died in two or three minutes.

"I plunged half the body of a leech into sp. of wine, and found in a little time that this part had lost all motion, whilst the other half continued in action. The experiment succeeded in the same way, whether the part of the leech toward the head was plunged or that towards the tail.

"The same consequences ensued on plunging a leech into the solution of opium in sp. of wine, or into the solution of it in water; and I looked upon it as something very extraordinary, that one half of the creature should become dead, whilst the other half continued in the state of not having undergone any change, or suffered any injury.

*Injections made into the Anus of Turtles.*

I injected three turtles of the same size, at the anus, by means of a glass syringe. On the first of them I tried sp. of wine, and in a few minutes it could scarcely stir. At the end of an hour it was quite dead.

"I injected the second with an equal quantity of a strong solution employed on *different* subjects; or while the matter in question is not acknowledged by both parties to be the same. They doubt whether any advocates for the *stimulant* doctrine would admit the first of the above definitions; and it appears that the advocates for the sedative opinion will not admit the definition given by C. C. W. therefore no legitimate dispute can exist between them at present.

solution of opium in sp. of wine, and in half an hour, it scarcely seemed alive or able to stir: It died at the end of the seventh hour, but the motions of the heart continued for an hour after.

"I injected the third with the same quantity of the aqueous solution (of opium): It was very lively at the end of the sixth hour, and survived the sixteenth.

"I have, however, observed in general, that turtles do not die when injected at the anus with the solution of opium in water. Those I injected with the spirituous solution, all died in less than three hours: *The injection was scarcely made when they lost their strength and vivacity, and in half an hour they almost ceased to give any signs of life.*"*

*Injection made beneath the Skin of Turtles.*

"I made an opening with a lancet in the skin betwixt the legs and belly of a turtle, and injected within it spirit of wine. *In a few seconds the turtle became motionless,* and died in less than an hour.

"I injected another turtle in the like way, with an equal quantity of the spirituous solution of opium. *In seven minutes it became without motion,* and died at the end of four hours."

"I injected a third with the aqueous solution of opium. It continued lively for two hours, but died at the end of the eighth.

"The result of a repetition of the same experiments on nine other turtles, was perfectly analogous to that of the foregoing."

*Turtles in which the Heart was laid bare.*

"I stripped the pericardium from the heart of a turtle, and applied to it sp. of wine several times successively. *In twenty minutes it had lost all motion, notwithstanding the animal continued alive. It died, however, in less than an hour,* WHEN NO PART OF ITS BODY WAS ANY LONGER IRRITABLE.

"I applied the spirituous solution of opium to the heart of another turtle, prepared as above. *In half an hour it was become motionless,* EVEN TO STIMULATION. The animal died at the end of three hours.

"I applied aqueous solution to the heart of another tur-

* I am sorry your Correspondent seems to have a dislike to italics and capitals, because I think them highly useful on many occasions; and on some, that they are indispensably necessary.

tle, and it continued its motion perfectly well for two hours: it still stirred a little at the end of the sixth. The turtle lived eight hours.

"I laid bare the heart of another turtle, and sprinkled it successively with several drops of sp. of wine. *The two auricles* INSTANTLY *ceased to move, and the heart* IN LESS THAN TWO MINUTES *had no longer any motion,* EVEN WHEN STIMULATED. *The turtle lived a long time in this state.*"

*Turtles, the Hearts of which were detached from the Thorax.*

"I removed the heart of a turtle from the thorax, and covered it with spirit of wine. *Its motion ceased in a few minutes.*

"I put the spirituous solution of opium on the heart of another turtle. In the space of a quarter of an hour there was scarcely any contraction of it, and in twenty-six minutes, it no longer stirred, *even when stimulated.*

"I plunged the heart of another turtle into the aqueous solution. It still continued to move, but not forcibly, at the end of half an hour. In two hours all was at rest.

"I plunged another heart into *simple water, and it still retained a little motion at the end of the third hour.*"

*Frogs injected beneath the Skin.*

"I injected a frog beneath the skin with sp. of wine. *It died in a minute after.*

"I injected a second with the spirituous solution of opium, and in a short time it could not support itself on its feet. It however was able to crawl a little at the end of thirty-five minutes, and died on the fortieth.

"I injected a third, with the aqueous solution of opium. In the space of ten minutes it scarcely stirred, and had its legs stiff and extended. It died in forty minutes."

*Hearts of Frogs separated from the Thorax.*

"I put the heart of a frog into sp. of wine. *Its motion ceased in two seconds.*

"I put a second heart into the spirituous solution of opium. *It ceased to move in twenty seconds.*

"I put a third into *pure water. It continued in motion for forty minutes.*

"I put the hearts of three frogs into pure water. The motion of one of them ceased in twenty-one minutes; *but it spontaneously recovered its oscillations, and that repeatedly.*

"The

"The second lost its motion in ten minutes, *but recovered it of itself.*

"The third ceased to move *at the end of fifty minutes**." Supplement to Fontana's Treat. on Pois. vol. 2. p. 364—374.

For the remainder of the experiments on this subject, see the last part of the Supplement, p. 375---395.

The following Experiments are taken from Dr. Alexander's Thesis.

CAPUT. I.

An Edit Effectus in Fibram Muscularem?

Sect. I.

"Solutio crudi opii, ad rationem unius drachmæ ad unam aquæ unciam, facta, et percolata, residuum in chartula unius scrupuli quatuorque granorum reliquit, ideoque triginta et sex grana opii tantum dissolvebantur. Hujus solutionis semuncia, ad temperaturam quadraginta et quatuor reducta, viz. ad rana temperaturam. Cujusdam ex istis animalibus vivi, thorace aperto, cor remotum vicies et quinquies in sexagesima parte horæ, se contraxit.

Experiment 1.

H. M.
12 0 "Cor in hujus solutionis immersum fuit semunciam.
0 2 Cordis contractiones multo minuebantur, quippe quæ jam quindecim haud superavere.
0 8 Contractiones destiterunt. Cor a fluido remotum mechaniceque stimulatum, nihil omnino contractionis ostendit.

Experiment 2.

12 20 Cor pari modo alia rana exscissum, in semunciam spiritus vini immersum, dum se ter decies in sexagesima horæ parte contraheret.
0 22 Prima immersione, tres vel quatuor contractiones potentes exhibuit, at exinde nullas. Fluido detractum, et arte etiam stimulatum, nullus tamen ciebatur motus.

Experiment

* The remarks afterwards made by Fontana would tend to weaken the effect of the above experiments, had they not been confirmed by other Experimentalists.

II. M. **Experiment 3.**

1 0 Cor tertiæ ranæ eadem operatione, ac primæ, exscissum, quod vicies et quater in sexagesima horæ parte contrahebat, in semunciam aquæ distil. temp. 44 immersum est.

0 2 Cordis contractiones post immersionem crebræ pariter ac antea fiebant.

0 16 Jam cessavere contractiones, cum corde remoto et vitro horarii sicco superimposito, mechaniceque irritato, contractiones per decimam horæ partem non destitere. Eadem experimenta cordibus cuniculorum repetita erant.

**Experiment 4.**

10 0 Cuniculi vivi mediæ magnitudinis conciso thorace, cor removebatur. In solutionis temperaturæ coeli unciam immersum, vicies et ter in sexagesima horæ partæ contrahebat.

0 4 Octodecim fiunt contractiones.

0 10 Sex vel octo in sexagesima horæ parte perlanguidæ contractiones.

0 12 Contrahi cessatum. Cor fluido eductum, mechaniceque irritatum, nihil tamen motus visum est. Hoc experimento sæpe repitito, cor unum spatio octo horæ momentorum quievit; alias tamen multas contractiones exhibuisse, post quindecim momentum, clare constabat.

**Experiment 5.**

11 0 Alius cuniculi cor in unciam *spt. vin. rect.* immersum.

0 2 Complures acres contractiones statim secutæ sunt, quæ jam haud parum defecere.

0 9 Nihil contractionis visum: cor eductum mechaniceque stimulatum, paucos languidos motus exhibuit.

**Experiment 6.**

11 30 Cuniculi vivi cor thorace exscissum, in aquam temperatura coeli immersum, quinquagies in momento horæ contrahebat.

0 34 Contractiones sæpe repetuntur.

0 40 Contrahebat languide; triginta tamen motus in sexagesima horæ parte numerantur.

0 50 Nihil motus visum. Cor remotum, et sicco vitro superimpositum mechaniceque stimulatum, per decem minuta contrahere perstitit."

The

The above experiments are, I presume, amply sufficient to shew, that I have at least made one assertion that is neither hasty nor unfounded; namely, that alcohol is a sedative; but I have several other experiments and arguments, at least as decisive, to advance in support of the assertion; but must defer them at present, fearing that I have already trespassed on the limits of your Journal.

I am, &c.

*Manchester, Dec. 9, 1802.* M. WARD.

